# The pandemic and changes in early career researchers’ career prospects, research and publishing practices

**DOI:** 10.1371/journal.pone.0281058

**Published:** 2023-02-15

**Authors:** Hamid R. Jamali, David Nicholas, David Sims, Anthony Watkinson, Eti Herman, Cherifa Boukacem-Zeghmouri, Blanca Rodríguez-Bravo, Marzena Świgoń, Abdullah Abrizah, Jie Xu, Carol Tenopir, Suzie Allard

**Affiliations:** 1 School of Information and Communication Studies, Charles Sturt University, Wagga Wagga, NSW, Australia; 2 CIBER Research, Newbury, Berkshire, United Kingdom; 3 School of Information Sciences, University of Tennessee, Knoxville, TN, United States of America; 4 Department of Computer Science, Université de Lyon, Villeurbanne, France; 5 Área de Biblioteconomía y Documentación, Universidad de León, León, Spain; 6 Wydział Humanistyczny, Uniwersytet Warminsko-Mazurski, Olsztyn, Poland; 7 Department of Library &Information Science, University of Malaya, Kuala Lumpur, Malaysia; 8 School of Information Management, Wuhan University, Wuhan, China; University of Manchester School of Biological Science: The University of Manchester Faculty of Biology Medicine and Health, UNITED KINGDOM

## Abstract

**Introduction:**

As part of the Harbnger-2 project, this study aimed to discover the impact of the COVID-19 pandemic on junior researchers’ work-life, career prospects, research and publishing practices and networking.

**Methods:**

An online international survey of 800 early career researchers (ECRs) was conducted in 2022. A questionnaire was developed based on three rounds of interviews and distributed using multiple channels including publishers, social media, and direct email to ECRs.

**Results:**

The impact of the pandemic on career prospects, morale, job security, productivity, ability to network and collaborate, and quality and speed of peer review has on the whole been more negative than positive. A quarter of ECRs shifted their research focus to pandemic-related topics and half of those who did, benefited largely due to increased productivity and impact. The majority worked remotely/from home and more than two-thirds of those who did so benefitted from it. While virtual or hybrid conferences have been embraced by the majority of ECRs, around a third still preferred face-to-face only conferences. The use of library online platforms, Sci-Hub, ResearchGate, Google Scholar and smartphone to search and access full-text papers increased. ECRs prioritised journals with fast submission procedures for the publishing of their papers and spent more time on increasing the visibility of their research. Fees were a problem for publishing open access.

**Conclusion:**

Although, generally, the pandemic negatively impacted many aspects of ECRs’ work-life, certain research areas and individuals benefited from being more appreciated and valued, and, in some cases, resulted in increased resources, better productivity and greater impact. Changes, such as the use of digital technologies and remote working created new opportunities for some ECRs. While continuing work flexibility and hybrid conferences might benefit some ECRs, institutions should also take measures to help those ECRs whose career and productivity have been adversely impacted.

## Introduction

Early career researchers (ECRs) are a large part of the research workforce [1,2] and a considerable range of scientific research is carried out with their help [3,4]. However, they are also vulnerable security-wise as they are likely to be graduate students, postdocs or on fixed-term/non-tenure contracts [5,6]. The literature leaves little doubt that ECRs have been disproportionally affected by the COVID-19 pandemic and they bear the brunt of the burden of the pandemic-incurred hardships [2,7–11]. Given that ECRs constitute the next generation of researchers, who will spearhead further developments, any changes in their research related attitudes and practices have great implications for the future of science. Thus, it is critical to understand the impact of the pandemic on ECRs’ work life, attitudes and practices, which is what we set out to do in the Harbingers-2 research project.

The extensive review of the literature on the topic [8], written on the eve of embarking upon the *Harbingers-2* project in the first year of the pandemic, focused on the challenges that were predicted to be or were already facing ECRs, culminating in the ‘horror scenario’ of their turning into a lost generation. By now, when the research environment in many countries is slowly re-assuming a semblance of normality, it seems quite clear that pandemic-era young researchers are far from having become a generation lost to the scientific endeavour [12]. Still, the trials they have encountered in the years of the pandemic certainly did not leave them unscathed.

A principal concern, repeatedly raised in the studies that looked at ECRs’ circumstances when the pandemic was at its height, was the likelihood of a negative effect on the career trajectory of newcomers to academe [8]. Indeed, as the most vulnerable cohort in the research community, ECRs were seen as particularly prone to the hiring freezes and layoffs resulting from the dire financial situation in Higher Education institutions, entailed mainly by the sharp drop in international student enrolments and the attendant loss in tuition fee and teaching grants, but also by cutbacks in external funding. The concerns did indeed turn out to be justified: for example, according to U.S. Labor Department estimates, colleges and universities closed out 2020 with continued job losses, shedding a net total of at least 650,000 workers since the World Health Organization declared a pandemic [13]. Indeed, in study after study ECRs report that their career prospects were significantly affected by the pandemic [8,11,14].

Hardly surprisingly, of course, with ECRs’ productivity, the key to a successful scholarly career [15–18], so often adversely affected by the lockdowns and social-distancing characterizing the first year or so after the onset of the pandemic. Indeed, researchers were faced with a host of challenges in their efforts to work in the manner they had been accustomed to: the suspension of many lab- and field- based research activities, the general shift to remote–and often more time consuming–working practices, the dearth of opportunities for networking and collaboration, the additional caring responsibilities, the all-pervading climate of stress and the COVID-associated physical and mental health problems, to name but the most frequently cited ones [7,9,10,19,20].

Moreover, even when the pandemic’s initial impacts were alleviated, some of its effects were found to persist; thus, for example, when the amount of time scientists were spending on their research had almost returned to pre-pandemic levels, they were still considerably less likely to pursue new research projects, a state of affairs that has been attributed to the massive shift during the pandemic to online scientific work [21]. Indeed, as it has been shown, virtual communications may not be as conducive to the formation of new ideas as face-to-face interactions [22].

Another outcome of the pandemic likely to leave long-lasting effects is the exacerbation of existing disparities among scientists. Indeed, a host of studies evidence the disproportionate impact of the pandemic on academic work by race, disability status, academic career stage, and, most notably, by gender [7–9,19,20,23–26]. As the relative change in the gender gap that occurred during the pandemic has been found to be the biggest for early-career scientists [27] as well as enduring between 2020 and 2021 [28], there can be little doubt that today’s ECRs need to be prepared for further challenges ahead. Judging from their reports in the interview stage of this study [12,29], they are up to the challenge.

What is presented here is the result of a global survey which was the last phase of Harbinger-2. The project, a mixed-method longitudinal study, included three rounds of interviews with a large cohort of ECRs in eight different countries (China, France, Malaysia, Poland, Russia, Spain, UK, and USA). The results of interviews are published elsewhere [12,29–31]. The last phase of the study was an international survey to test and triangulate some of the findings of the interviews on a larger audience. The survey specifically sought to answer these questions:

- To what extent were ECRs’ career prospects, job security, morale, research productivity and research focus impacted by the pandemic?
- How widespread was working remotely/from home and did this benefit ECRs or disadvantage them?
- To what extent were some scholarly information practices (use of information services, for instance) impacted by the pandemic?
- What changes have occurred in the publishing practices of ECRs?
- To what extent did the pandemic influence ECRs’ attitudes towards research integrity and quality?
- What were the preferences for and attitudes of ECRs towards networking and attending conferences (online/ in-person/ hybrid)?

## Method

A questionnaire was developed based on the outcome of the qualitative phases of the study (three rounds of interviews) and piloted. The questionnaire included 17 questions about the impact of the pandemic on scholarly communication practices and attitudes, as well as a few demographic questions. The questions were largely based on the issues that emerged from the interviews, which we wanted to test on a larger and more diverse population. The questionnaire was translated by the research team into Chinese, French, Polish and Spanish and was hosted on Qualtrics in the third quarter of 2022. It should be said that Russia was also initially part of the project but was dropped due to problems resulting from the war in Ukraine.

For the sake of consistency and to be able to triangulate the data, we used the same definition for ECR (below) used in the other phases of the Harbinger2 project. The survey started with a screening question that asked respondents to self-identify whether they were an ECR based on the definition. Those who said *No* in answer to the screening question exited the survey.

*“We are most interested in hearing from researchers who are generally no older than 45, who either have received their doctorate and are currently in a primarily research position or have been in research positions but are currently doing a doctorate. In neither case should researchers be in established or tenured position. But if all of that is just too complex and if you believe you are an early career researcher that is all that counts!”*

### Sampling

We did not have a sampling framework because there is no register of ECRs in any of the case study countries. Therefore, a probability sampling approach was not possible, and we decided to distribute the survey as widely as possible through various channels. Four methods were used for distribution:

- Invitations were sent out by scholarly publishers or relevant institutions to potential ECRs (e.g., Taylor and Francis).
- A link to the survey was tweeted by publishers or relevant institutions to researchers (e.g., Oxford University Press).
- A banner image with a link to the survey was put on Wiley Digital Library and anyone who saw the banner while visiting a journal or article and was interested could click and go to the survey.
- Direct invitation emails and texts were sent to ECRS at universities in the case study countries by the national interviewers.

### Characteristics of respondents

After data cleaning, 800 responses remained for analysis. To comply with research ethics, respondents were allowed to skip any questions they did not want to answer.

As Table 1 shows, respondents included slightly more women (440, 55%) than men (314, 39.3%). The majority had a doctorate (493, 61.6%), and were 31 years or older (560, 70.1%). The disciplinary distribution of respondents was not even, with the largest group coming from social sciences (294, 36.8%) and the second largest coming from life/biological sciences (158, 19.8%). Chemical sciences (21, 2.6%) and mathematical sciences (28, 3.5%) had the lowest numbers of respondents. Respondents were researchers working in 71 countries. Those based in the USA accounted for slightly more than a third of responses (285, 35.6%), followed by a large gap to China (61), Spain (48), France (40), Australia (31), Malaysia (30), India (28), UK (27) and Poland (17). We also asked whether respondents had any caring responsibility (for children or family members, for instance) and a little less than half (353, 44.1%) said they did. About half (50.6%) of those with a caring responsibility were women as compared to 46% that were men, and the rest had another gender). Those with caring responsibilities were more likely to be in the older age category, thus, 59% of them were 36 years or older while only 21% of those without caring responsibilities were 36 years or older. Comparing the make-up of the survey respondents with those who participated in the interview phase of the study, the survey respondents were on average older, and they included arts and humanities ECRs which were not part of the interview cohort. In addition, the health/medical sciences were the largest disciplinary group in interviews, whereas they were the third largest group in the survey.

**Table 1:** Demographics of respondents.

Demographics	Items	N	%
**Gender**	Woman	440	55.0
	Man	314	39.3
	Other, prefer not to say, self-describe	29	3.6
	No answer	17	2.1
**Age**	Under 20	1	0.1
	21–25	44	5.5
	26–30	174	21.8
	31–35	263	32.9
	36–40	166	20.8
	41–50	131	16.4
	No answer	21	2.6
**Degree**	Bachelor’s degree	43	5.4
	Master’s degree	228	28.5
	Doctorate degree	493	61.6
	Professional degree	16	2.0
	Prefer not to say	3	0.4
	No answer	17	2.1
**Subject category**	Mathematical sciences including computer science	28	3.5
	Physical sciences including engineering/technology	56	7.0
	Chemical sciences	21	2.6
	Life/biological sciences including agriculture	158	19.8
	Health/medical sciences	141	17.6
	Environmental sciences	40	5.0
	Social sciences	294	36.8
	Arts and humanities	43	5.4
	Other, please	3	0.4
	No answer	16	2.0
**Caring responsibility**	Yes	353	44.1
	No	431	53.9
	No answer	16	2.0

### Data analysis

Statistical analysis including descriptive (frequency and percentage) and some inferential (non-parametric Chi-square, Mann Whitney U, and Kruskal-Wallis H tests) were conducted using the Statistical Package for the Social Sciences (SPSS). Non-parametric tests were used because of the nature of variables (some nominal or ordinal) and lack of normality of the data. For six questions with Likert options (strongly disagree to strongly agree, or significant negative impact to significant positive impact) the mean value was also calculated using numeric values of the options (1 being ’strongly disagree’ or ’significant negative impact’ and 5 being ’strongly agree’ or ’significant positive impact’). ’Don’t know’ or ’not sure’ options were excluded in the mean calculation for these questions. We have reported demographic differences throughout the findings where such differences are statistically meaningful. For gender, only differences between men and women were examined (excluding n = 29 who were non-binary, self-described or preferred not to say). Since the number of respondents in some demographic variables (some subjects or countries) were small, Monte Carlo p value was used instead of asymptotic. Comparisons between countries were only made in the case of seven countries that were included in the qualitative phase of the study, so that we could compare the survey findings with interviews. They were China, France, Malaysia, Poland, Spain, UK, and USA. Open ended questions were analysed using thematic coding. Where we have used quotes from respondents, we have mentioned gender, subject and country if known in square brackets.

The survey data (csv file) and the questionnaire are available as a dataset on Figshare [32].

### Ethics statement

Ethics approval for this survey was given by the University of Tennessee Knoxville’s Institutional Review Board (IRB) (approval number = UTK IRB-22-06930-XP). Implied informed consent was obtained from all participants. To do this, the first page of the survey was the consent page which included information about the research scope, and that their answers were confidential, and no respondent would be identifiable in the presentation of the results. This page ended with a statement for participants to first acknowledge reading and understanding the informed consent before entering the survey. Informed consent was not written or verbal, instead participants gave their implied informed consent by clicking on "I Agree" button before the survey began. Those who clicked on “I Do Not Agree” were pushed out of the survey with a thank you note. The consent page stated that “you must be age 18 or older to participate in the study” and no data was collected from people under 18.

## Findings

### Impact of the pandemic on career

**Fig 1:**
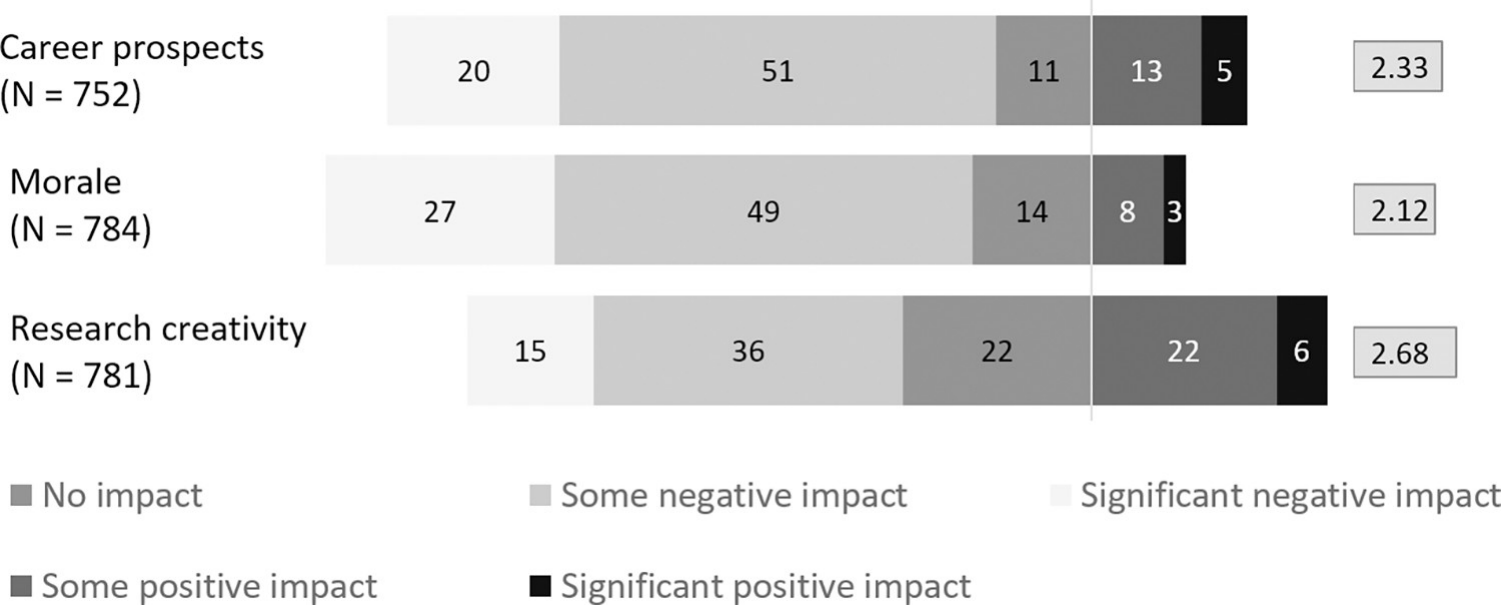
What overall impact do you think the pandemic has had on your…?

Respondents were asked about the overall impact of the pandemic on their career prospects, morale, and research creativity. The divergent stack bar (Fig 1) shows the percentages of responses as well as the mean value of the Likert scales. Overall, the impact of the pandemic on the three aspects was negative as all mean values are below 3 (expected average). However, the negative impact was less severe for research creativity and most severe for morale as can be seen from both mean values and percentage of negative or positive impacts. Twenty-eight per cent of respondents saw a significant positive or some positive impact on their research creativity while the figure for morale was just 11%. Some statistically significant differences were found for gender and subject. Thus, the impact on morale was more negative for women (M = 2.07) compared to men (M = 2.2) (H = 5.4, df = 1, p = .02), and the impact on career prospects was most negative for chemistry (M = 2) and least negative for the environmental sciences (M = 2.7) (H = 18.5, df = 7, p = .01).

**Fig 2:**
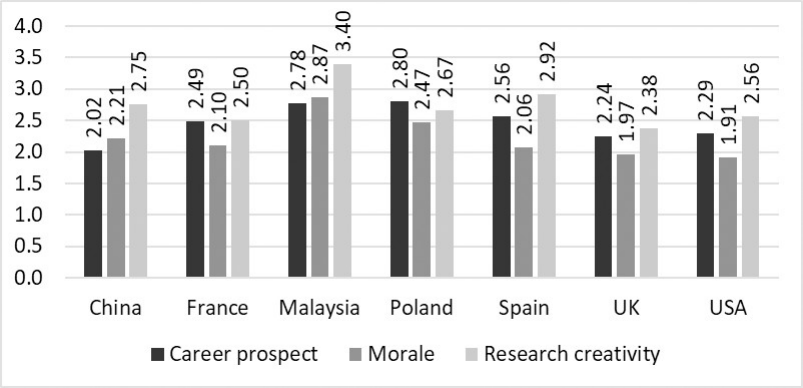
Country differences for overall impact.

There were also differences between the seven countries (Fig 2). The impact on career was most negative for China and least negative for Malaysia (H = 15.3, df = 6, p = .01). The impact on morale was most negative for the USA and least negative for Malaysia (H = 36.1, p = .001). The impact on creativity was most negative for the UK and least negative for Malaysia (H = 20.1, df = 6, p = .003).

A free-text question gave those who chose some or significant negative or positive impact (excluding those who said no impact or don’t know/not sure) an opportunity to explain *how* the pandemic impacted their career prospects. Out of those who said a significant negative impact or some negative impact, 465 left a comment. The dominant theme for this group was a delay in research activities which merited 155 comments. It was variously said that labs were closed, field work was not possible, the purchase of material and instruments was slow and difficult, institutions shut down for some time and conducting certain types of research was either unfeasible, very difficult or simply not high in the priority list, and therefore, not well supported. Many researchers lost a few months of their time with a delay in data collection or other research activities. All of this meant a drop in productivity. The first quote below provides an example of a delay in research and the consequential drop in productivity. Reduced productivity was in fact mentioned by 34 ECRs. Due to these challenges some ECRs had to shift their research focus and choose topics or areas that were not their real interest and not what they necessarily wanted to study. The second quote below shows an example of a shift in research topic.

*I started my faculty position in 2019*. *My research takes place in elementary schools*, *and I was not able to collect any new data while schools shut down*. *My start-up funds were not able to be used*. *Meanwhile*, *colleagues who do work online or with college students kept working*, *leaving me comparatively farther behind in terms of productivity*. *This has also made me uncompetitive for grants*, *awards*, *and raises* (man, social sciences, USA).

*During 2020–2022*, *my research was forced to shift to computational work that I could do at home*. *This was not my interest area and resulted in me missing opportunities to gain experience with wet lab techniques that I will need for my future career*. *Overall*, *I feel that I’ve fallen behind and I don’t have the skillset that most of my peers (later stage PhD students) do* (woman, health/medical sciences, USA).

Job insecurity was the second most dominant theme with 136 comments being related to it one way or another. They included loss of job opportunities due to a delay in entering the job market, hiring freezes, travel restrictions, contracts not being renewed, undesirable changes in career trajectory, and difficulty in securing tenure due to changes in work conditions.

The third major theme was related to loss of interaction, mostly face-to-face and therefore, impacting on the inability to network and collaborate. Eighty comments were related to this. Isolation (12) was another problem that was related to the reduced opportunity to interact, network, and collaborate. All such problems can damage the motivation (14) and create mental health issues (8). Stress (14), anxiety (7), depression (5), and fatigue (4) were some of the issues mentioned by respondents. The quote below illustrates how lack of interaction resulted in isolation for an ECR.

*I joined a department as an assistant professor during the pandemic and ended up very isolated*. *Because of the pandemic*, *there were no welcome events*, *no training*, *no mentorship*, *no social events*. *No one invited me to collaborate*. *I didn’t meet most of the faculty in my department until I had been here nearly two years* (woman, life/biological sciences, USA).

With economic difficulties that caused by the pandemic in all countries, research funding was cut or shifted to areas that were considered higher in the funders’ priority (usually pandemic-related research). This meant that it became more difficult to get funding for many ECRs due to funding shortage and increased competition. Funding was mentioned by 43 respondents.

Working from home did not work for everyone. For some this meant new responsibilities such as home schooling and caring that would come at the expense of research time. Some others simply found it difficult to work from home (e.g., due to distraction etc.) and all of these groups became less productive. Challenges associated to working from home was mentioned by 19 respondents.

*I had children at home and getting research done was difficult while trying to get their school also done*. *In addition*, *shift to remote learning took more effort than delivering in person classes* (man, social sciences, USA).

From those who mentioned some positive impact or significant positive impact, 118 left comments. They mentioned the pandemic gave them more time to write papers (11), more time to spend on research or focus on research (6), and more time to reflect on their career (4). Working from home or remotely positively impacted some (15) as they could spend more time with family and improve their mental health or avoid wasting time in meeting or admin tasks. The pandemic also meant more collaboration opportunities (8) or more funding (6) or more research opportunities (6). For those in certain fields that had some significance for the pandemic related research such as microbiology, epidemiology, biostatistics and so on, the pandemic meant that their research was more in demand and more acknowledged and appreciated (12). The pandemic created new job or career opportunities for some of this group (23) and this happened for various reasons including the decision by many senior academics to retire or increased demand in certain fields. The pandemic also gave opportunity to some to learn new skills and do some personal and professional development (4). Increased productivity (5), better or more focus on mental health (3), more impactful research (3), more online conference and seminar attendance (3), and ability to finish their PhD (6) were other positive impacts that some mentioned. Some example quotes are presented below.

*The pandemic facilitated a career transition from basic science to clinical applied science*. *Following the transition my individual career prospects have expanded* (man, health/medical sciences, USA).

*I’m an epidemiologist*, *so my research and my field of expertise is now more valuable and appreciated* (unknown gender & subject, Colombia).

*Yes*, *my mental health improved because I could work from home*. *It was Easier to join meetings because I was not racing between buildings on campus*. *I was able to do more focused work at home and not commute in or balance too many things* (woman, health/medical sciences, USA)].

### Job security

The pandemic created job insecurity for many researchers. Slightly more than a third of respondents (37.6%) felt less secure in their employment situation compared to pre-pandemic and only 12.3% felt more secure (Table 2).

**Table 2:** How secure do you feel in your employment situation compared to pre-pandemic times?

	All		Women		Men	
Job security	N	%	N	%	N	%
Less secure	300	37.6	147	36.1	134	40.1
Same	344	43.1	198	48.6	132	47.1
More secure	98	12.3	62	15.2	28	12.8
Don’t know/ not sure	56	7.0	-	-	-	-
Total	798	100	407	100	294	100

There was a gender difference as men were more likely to say they felt less secure (45.6% of men vs 6.1% of women) and women were more likely to say they felt more secure (15.2% of women vs 9.5% of men) (*X*^*2*^ = 8.65, df = 2, p = .01). There were no differences between countries and other demographic attributes.

### Changes in research focus

We asked respondents if they changed course in their research to do pandemic-related research and the majority (574, 71.8%) said *No* their research has not been pandemic-related and they did not shift to pandemic-related research. Another five per cent said they did not change because their research was already pandemic related, and finally 185 (23.2%) said they did change their research focus to do pandemic-related research. Those who did not have caring responsibilities were slightly more likely to change their research focus (25.5%) compared to those without caring responsibility (20.5%) (*X*^*2*^ = 7.3, df = 2, p = .02).

**Fig 3:**
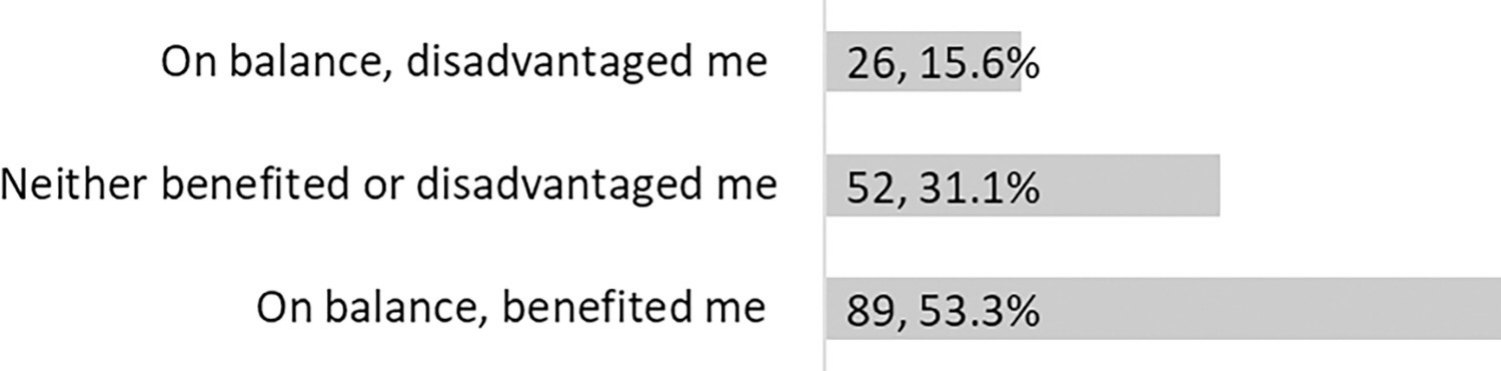
If changed research focus, has this change benefited or disadvantaged you in any way?

Those who changed their research focus were asked if the change benefited or disadvantaged them in any way and 167 responded (Fig 3). For the majority (53.3%) this change has resulted in benefits and only for 15.6%, the change disadvantaged them.

We also gave these respondents a free text space to explain why they benefited or were disadvantaged. Thirty-eight who were disadvantaged left comments. The main issue for them seemed to be that the change of focus resulted in a need for learning new skills. Some had to start from square one and some others had to change their research design. For some, their research was delayed or slowed down due to factors, such as a lack of physical connection to the research population or stakeholders, or an inability to do in-lab research. ECRs like many other researchers, normally follow their research interests. However, the shift in research focus for some meant that they had to research a topic that was not their real interest. This resulted in a feeling of underperforming and a worry about competing in an even more competitive area which was not their primary area of research and strength. Examples of comments are:

*Shifting focus suddenly and through external pressure during a very emotionally-volatile time so early in my career has certainly set me back in terms of productivity approximately 2 years* (woman, social sciences, USA).

*I was not actually interested in pandemic-related research*, *but that is the direction many of my collaborators and funders wanted to move in*. *Lack of interest in the research led to lack of motivation*. *Additionally*, *many people were switching to research on these topics*, *and I believe there was a certain level of fatigue that set in*, *in terms of hearing about this research*, *either at conferences or in publications*. *It has been difficult to get the research published* (woman, social sciences, USA).

Moreover, the changes acted like a stress test as it put a lot of pressure on ECRs, which resulted in once hidden problems resurfacing in their lives. As one ECR put it “*It’s hard to say because I am still suffering through it*, *but my experience during the pandemic exposed unhealthy aspects of my life that I had feared and avoided for a long time*. *Hopefully*, *it will turn into a catalyst for growth”* (man, chemical sciences, USA).

Ninety-two of those who said they benefited also left a comment. Most of their comments concerned research productivity, impact, collaboration, and growth. The most mentioned benefit was in fact productivity, that is being able to publish more papers, and not just any papers, papers that were well-received and cited. They mentioned that generally COVID related research is well cited as the comment below demonstrates:

*The papers on Covid-19 get cited quite frequently*, *which benefits our h-index*, *which is still used in hiring decisions and by grant reviewers to rank applicants* (woman, social sciences, Switzerland).

Greater productivity was also linked to other factors, such as, obtaining more grant opportunities, more collaborations, obtaining new research topics, increased opportunities for cross-disciplinary research, the faster pace of research, and access to data that they otherwise would not have had. Some others benefited because their research area (e.g., public health, health communication, epidemiology) gained prominence during the pandemic. Their research was “*timely and easily publishable*”. Other ECRs benefitted because they already had research skills that were in high demand (e.g., certain lab skills or statistics) or they found the opportunity to learn such new skills, and therefore, new opportunities emerged for them either as new employment or increased their reputation and visibility. One for instance said: “g*ot some coverage in my field through my pandemic-related projects*” (man, health/medical sciences, USA). A few also stepped up and took more responsibilities in their team or established their own team.

There were other benefits too. One ECR mentioned the progress open science made during the pandemic. Two mentioned that they were able to save time and money: “*from a work standpoint*: *saved money on commuting*, *lunch; fewer meetings; quiet home environment to work from*” (man, life/biological sciences, USA). For a few, their research was more easily doable in an online environment (e.g., doing interviews). And finally, a few were looking at a glass half-full, being happy that they “*were able to do research when many others weren’t*” (woman, social sciences, USA) or because it “*gave {them} something to pour {their} energy into during a very scary time*” (woman, social sciences, Denmark).

### Working from home

Most countries went through a period of lockdown during the pandemic, which resulted in academics and researchers working from home or employers making working conditions more flexible and allowing remote working. We wanted to know to what extent this was the case for ECRs and whether they saw it as a hindrance or benefit (See Table 3). Only 11.4% did not work from home or remotely and in terms of its effect, the response was mixed as might have been expected. While more than a third (39.1%) benefited from working from home, another third (35.8%) saw it more as a hindrance and for the remaining, it was neither a benefit nor a disadvantage.

**Table 3:** Was working from home and/or working remotely a benefit or a hindrance?

	All		Women		Men	
Working from home	N	%	N	%	N	%
I didn’t really work much from home or remotely	91	11.4	36	8.2	48	15.4
On balance a benefit rather than a hindrance	311	39.1	189	43.1	104	33.4
On balance a hindrance more than a benefit	285	35.8	149	33.9	119	38.3
On balance, neither a hinderance nor a benefit	109	13.7	65	14.8	40	12.9
Total	796	100	439	100	311	100

Men were more likely not to work from home/or remotely (15.4% of men vs 8.2% of women) or to see it as a hindrance (38.3% of men vs 33.9% of women), and women were more likely to find it a benefit on balance (43.1% of women vs 33.4% of men) (*X*^*2*^ = 14.25, df = 3, p = .003). Age-wise, the younger the respondents the more likely they were to see it as a hindrance (63.4% of 21–25 age group vs 33.6% of 41–50 group saw it as a hinderance); vice versa so older groups were more likely to see it as a benefit (48.7% of 41–50 vs 24.4% of 21–24 saw it as a benefit) (*X*^*2*^ = 30.29, df = 12, p = .003). Surprisingly, there was no difference between those with and those without caring responsibilities. Although the responses to this question varied by country, for instance, the Chinese had the highest percentage of ECRs benefiting from working from home (50%) and the Polish had the highest percentage seeing it as a hinderance (58.8%), although the differences were not statistically significant (*X*^*2*^ = 26.4, df = 18, p = .09).

### Research time

While the time devoted to research for 222 (28%) respondents had not changed as compared to before the pandemic, a large proportion 333 (42%) dedicated less time to their research than before the pandemic and 242 (30%) dedicated more time to research than before the pandemic (Table 4).

**Table 4:** What impact has the pandemic had on the time you allocated to research?

	All		Mathematical sci.		Physical sci.		Chemical sci.		Life/ biological sci.		Health/ medical sci.		Environmental sci.		Social sci.		Arts and humanities	
	N	%	N	%	N	%	N	%	N	%	N	%	N	%	N	%	N	%
Dedicated more time	242	30	10	37	16	30	8	38	37	25	39	29	11	28	88	31	17	43
Dedicated less time	333	42	6	22	30	56	10	48	62	42	59	43	13	33	119	42	13	33
Research time didn’t change	222	28	11	41	8	15	3	14	50	34	38	28	16	40	74	26	10	25
Total	797	100	27	100	54	100	21	100	149	100	136	100	40	100	281	100	40	100

ECRs from the physical sciences formed the largest percentage of those who said they dedicated less time to research (56%), and they were followed by those from the chemical sciences (48%) (Table 4). The highest percentage of those who said that they dedicated more time were from arts and humanities (43%) and then from the chemical sciences (38%). The largest percentage saying there was no change in research time devoted were from the mathematical sciences (41%) and environmental sciences (40%) (*X*^*2*^ = 30.3, df = 16, p = .005). Quite expectedly, those with caring responsibilities were more likely to dedicate less time to their research than before the pandemic (47.9% of those with vs 37.2% of those without caring responsibility) (*X*^*2*^ = 9.02, df = 2, p = .01), and those without caring responsibilities were more likely to dedicate more time to their research (32.6% of those without vs 27.4% of those with caring responsibilities). There was no gender or age difference. As illustrated in Table 5, there were significant differences between the seven countries (*X*^*2*^ = 29.4, df = 12, p = .003). Malaysia had the highest percentage of those who dedicated more time (16, 53.3%), Poland had the highest percentage of those who dedicated less time (9, 52.9%) and the UK had the highest percentage of those saying there was no change in research time (13, 48.1%).

**Table 5:** Country differences for change in research time.

		I dedicated more time to research than before pandemic	I dedicated less time to research than before pandemic	My research time did not change	Total
China	N	22	20	19	61
	%	36.1	32.8	31.1	100
France	N	13	17	10	40
	%	32.5	42.5	25.0	100
Malaysia	N	16	9	5	30
	%	53.3	30.0	16.7	100
Poland	N	2	9	6	17
	%	11.8	52.9	35.3	100
Spain	N	20	14	14	48
	%	41.7	29.2	29.2	100
UK	N	4	10	13	27
	%	14.8	37.0	48.1	100
USA	N	66	135	84	285
	%	23.2	47.4	29.5	100

### Research related activities

Besides time dedicated to research, we were interested in learning about changes in research-related activities. Table 6 presents the percentage of responses (and mean values) for whether the pandemic increased or decreased some specified scholarly activities related to research. These activities were selected on the basis of the findings of the interviews, trends, for instance, that could be checked on a larger scale. For the majority of ECRs (62.6%), the use of the library to access full-text papers did not change, but for more than a quarter (28.6%) it increased, perhaps because they spent more time writing papers and therefore, needing to read more papers too. However, the use of the other library services (such as borrowing books, inter-library loans etc) saw a smaller increase (15.5%) and it was more likely that the full-text article service not to have been used (15.3% said never used) or decreased (10.5%). Almost a quarter of respondents (24.1%) said their use of Sci-Hub increased and slightly more than a quarter (27.7%) said they had never used Sci-Hub to access full text of papers. While the use of ResearchGate for accessing full text of papers increased for 23.8% of ECRs, the increase for making their own articles freely available was lower (18.2% of ECRs). Interestingly, the percentage of those who said they had never used a repository to make their preprints accessible (36.5%) was higher than those who never used ResearchGate for such a purpose (25.3%). For most respondents, the use of Google Scholar to locate and access full text of articles either saw no change (62.1%) or increased (29.4%). Using smartphones to search for papers or to access them saw the highest percentage of ECRs who said they had increased their use (32%) and the respondents who increased their use of smartphones tended to be of younger age groups compared to those whose use decreased or did not change.

**Table 6:** What increasing or decreasing effect has the pandemic had on the following aspects of your work? (%).

No	Item statements	Never used	Stopped using	My use decreased	No change	My use increased	Total	Mean
**1**	Using library’s online platforms to access full-text of papers	4.0	1.3	3.5	62.6	28.6	797	4.11
**2**	Using the other services offered by the library (apart from accessing articles)	15.3	5.6	10.5	53.1	15.5	791	3.48
**3**	Using Sci-Hub to access full-text of papers	27.7	1.4	2.8	44.0	24.1	793	3.35
**4**	Using ResearchGate to access full-text of papers	14.4	1.1	3.4	57.3	23.8	791	3.75
**5**	Using ResearchGate to make your articles freely available	25.3	1.6	2.0	52.8	18.2	791	3.37
**6**	Using preprint repositories /or servers to make preprints of your papers freely available	36.5	1.3	1.9	45.5	14.8	791	3.01
**7**	Using Google Scholar to locate and access full-text of papers	4.9	0.9	2.8	62.1	29.4	796	4.10
**8**	Using smartphones to search for papers and/or access full-text of papers	15.2	0.9	2.3	49.6	32.0	796	3.82

There were some demographic differences. Men were more likely to have increased their use of Sci-Hub (31.9% of men vs 19.2% of women; *X*^*2*^ = 23.5, df = 4, p = .001) and ResearchGate to access full text of papers (31% of men vs 19.8% of women; *X*^*2*^ = 14.8, df = 4, p = .005). In the case of using library’s online platforms to access full text of papers, women were more likely to say there was no change (68.5% of women vs 54.6% of men; *X*^*2*^ = 20.5, df = 4, p = .001) and men were more likely than women to have never used, stopped using, increased using or decreased using the platforms. Subject-wise, there were some differences in regard to the use of preprint repositories, Google Scholar, and smartphones. The percentage of those who increased their use of repositories was highest among chemical sciences (28.6%). Arts and humanities (51.2%), social sciences (41.4%) and health/medical sciences (40.3%) had the largest percentages of those who said they have ‘never used’ repositories. The use of Google Scholar increased for 37.5% of environmental scientists and 32.9% of social scientists; and it was arts and humanities which recorded the largest percentage of ECRs who had never used Google Scholar (16.3%). The use of smartphones increased the most in the chemical sciences (52.4%) and environmental sciences (37.5%). The largest never used percentage for smartphones was recorded by the social sciences (19.5%) and arts and humanities (18.6%). Those who had caring responsibility compared to those who did not were more likely to be among those who said they increased their use of ResearchGate (28.2% of those with vs 20.3% of those without caring responsibilities; *X*^*2*^ = 18.9, df = 4, p = .001), Sci-Hub (25.9% of those with vs 22.4% of those without caring responsibilities; *X*^*2*^ = 18.9, df = 4, p = .001) and smartphone (37.1% of those with vs 27.4% of those without caring responsibilities; *X*^*2*^ = 15.1, df = 4, p = .004) to access full text of papers.

There were significant differences between the seven countries for all of the activities except the second one (use of the other services offered by libraries). Chi-square test results were significant at p < 0.05 for the other seven activities. The percentage of ‘my use increased’ for all items, except for the use of preprint repositories, was highest for Malaysian ECRs. France had the highest percentage of ECRs saying they never used library services to access full-text articles (7.5%), other library services (25%), Google Scholar (10%) and smartphones (25%). The highest percentage of ECRs having ‘never used’ for Sci-Hub were those from the US (40.7%) and UK (42.3%) and at the other end of the spectrum the countries with highest increase in use of Sci-Hub were Malaysia (40%), Spain (37.5%) and France (37.5%). China had the highest percentage of people saying they ‘never used’ ResearchGate for accessing papers (25%) and posting papers (38.3%). Preprint repositories were the least used in China (53.3% never used), Poland (52.9%) and the USA (43.1%). Preprint repositories recorded the highest increase in use in the UK (30.8%) followed by Malaysia (30%). It should be noted that in the UK the use of repositories is mandated by the UK’s national research assessment.

### Publishing practices

Table 7 presents the percentages and mean values of agreement/disagreement of respondents with some statements concerning their publishing practices. Similar to the previous question, these statements were based on the key findings of the interviews. The highest agreements were for article processing charges (APC) being a problem for publishing open access (OA) (M = 3.24) followed by prioritising journals with faster processing speed (M = 3.23). The issue with APCs might be because ECRs do not have access to funds as much as well-established researchers do. The lowest agreements (or highest disagreement) were related to the increase in productivity because of the pandemic (M = 2.70) and prioritising publishing OA as a result of the pandemic (M = 2.90).

**Table 7:** Think of your current publishing practices in comparison with practices before the pandemic and tell us to what extent you agree or disagree with each statement (%).

No	Item statements	Strongly disagree	Somewhat disagree	Neither agree nor disagree	Somewhat agree	Strongly agree	Total	Mean
**1**	My productivity (the number of papers I published) increased as a result of the pandemic	24.0	19.6	27.8	19.7	9.0	792	2.70
**2**	I prioritised publishing my papers as open access (either in OA journals or in Hybrid journals) as a result of the pandemic	13.9	11.6	51.7	16.0	6.8	793	2.90
**3**	I put more effort into ensuring my research is understandable to a wider audience as a result of the pandemic	9.6	11.8	46.5	21.4	10.8	790	3.12
**4**	Article Processing Charges or Open access publication fees were a problem for me when it comes to publishing open access papers as a result of the pandemic	10.4	8.0	45.1	19.8	16.6	787	3.24
**5**	I prioritised journals with higher impact factors when deciding where to publish as a result of the pandemic	9.7	11.2	50.0	19.1	10.1	786	3.09
**6**	I prioritised journals with faster processing speed when deciding where to publish as a result of the pandemic	9.3	10.1	41.6	26.3	12.8	784	3.23
**7**	I prioritised journals indexed in the Web of Science and /or Scopus when deciding where to publish as a result of the pandemic	12.2	7.9	55.0	13.0	12.0	786	3.05
**8**	I spent more time on activities that increase the visibility of my research (e.g. tweeting a link to my article, presenting at a conference, seminar or workshop (including virtual), writing in a professional magazine or a research newsletter, blogging, etc.) as a result of the pandemic.	11.7	12.5	36.3	27.6	11.9	787	3.16

Splitting the respondents to two age groups of those 35 years and younger, and those 36 years and older, there were significant differences for statements 6 (faster journals) and 7 (indexed journals) and in both cases the older group had more agreement with the statements. For the first three statements, the difference was small and not statistically significant. Subject differences existed for two statements. The one on productivity had the highest agreement amongst environmental sciences (M = 3.03) and the least agreement among chemical sciences (M = 2.30) (H = 20.2, df = 7, p = .005). Prioritising publishing OA had the highest agreement among chemical scientists (M = 3.48) and the lowest agreement among physical scientists (M = 2.59) (H = 16.6, df = 7, p = .02). Table 8 shows the mean value of the items for the seven countries and the differences are significant except for statements 2 and 8. US ECRs were more negative about the impact of the pandemic on their productivity and Malaysians were most positive about this. APCs were a bigger problem in Spain and the UK.

**Table 8:** Country differences for publishing practices.

	Practices	China	France	Malaysia	Poland	Spain	UK	USA
**1**	*My productivity increased	2.69	2.43	3.43	2.76	3.02	2.42	2.39
**2**	I prioritised publishing my papers as open access	2.84	2.90	3.10	2.94	2.79	3.00	2.71
**3**	*I put more effort into ensuring my research is understandable	3.13	3.08	3.70	2.65	2.98	2.62	2.98
**4**	*APCs were a problem for me when it comes to publishing OA	3.25	3.15	3.97	3.71	2.87	2.88	3.13
**5**	*I prioritised journals with higher impact factors	3.11	2.75	3.70	3.76	3.23	2.73	2.90
**6**	*I prioritised journals with faster processing speed	3.46	2.83	3.77	3.35	3.19	3.08	3.14
**7**	*I prioritised journals indexed in the Web of Science and /or Scopus	3.13	2.73	3.73	3.76	3.19	2.85	2.68
**8**	I spent more time on activities that increase the visibility of my research	3.20	3.08	3.67	3.35	3.04	2.96	3.04

* Significant at *p* < 0.01.

Men had more agreement with all statements (except the two statements about APC and prioritising publishing OA) than women and the difference was statistically significant. Table 9 shows the mean value of agreement/disagreement for men and women and for those with and without caring responsibilities. The agreement with statements 4 to 8 (from APC statement onwards) was higher for those with caring responsibilities compared to those without and the difference are statistically significant.

**Table 9:** Mean value of the agreement with statements by gender and caring responsibility.

No	Statements	Caring responsibility		Gender	
		Yes	No	Men	Women
**1**	My productivity increased	2.67	2.73	2.85*	2.64*
**2**	I prioritised publishing my papers as open access	2.95	2.86	2.93	2.89
**3**	I put more effort into ensuring my research is understandable	3.21	3.04	3.24*	3.04*
**4**	APCs were a problem for me when it comes to publishing OA	3.43*	3.09*	3.28	3.24
**5**	I prioritised journals with higher impact factors	3.23*	2.97*	3.25*	3.01*
**6**	I prioritised journals with faster processing speed	3.43*	3.08*	3.35*	3.21*
**7**	I prioritised journals indexed in the Web of Science and /or Scopus	3.24*	2.89*	3.24*	2.95*
**8**	I spent more time on activities that increase the visibility of my research	3.29*	3.04*	3.26*	3.09*

* Significant at p < 0.05.

We wanted to know about ECRs’ experience concerning the impact of the pandemic on the quality and integrity of research (Table 10). We defined integrity as conducting research that people can have confidence and trust in its method and results. Post-publication commenting on social media was the only aspect that they thought the pandemic has slightly affected positively overall as the mean value was 3.30 (above the expected average of 3) and more respondents chose positive options (33.8%) than negative options (11.2%). The impact on the research integrity was not so positive or negative either (M = 3), with 20.7% choosing positive impact and 23.6% choosing negative impact. However, the impact of the pandemic on the quality/standard of peer review (M = 2.85) and speed of peer review (M = 2.48) based on ECRs personal experience was more negative than positive. The only demographic difference was that those with a caring responsibility were more positive about the impact of the pandemic on research integrity (M = 3.09 for those with vs M = 2.94 for those without caring responsibility; U = 56048, p = .01) and quality/standard of peer review (M = 2.97 for those with vs M = 2.72 for those without caring responsibility; U = 52409, p = .002) than those without caring responsibility.

**Table 10:** Based on your personal experience, how has the pandemic affected the following in your field? (%).

Research quality/integrity aspect	Significant negative impact	Some negative impact	No impact	Some positive impact	Significant positive impact	Total	Mean
**Research integrity**	4.8	18.8	55.7	12.6	8.1	724	3.00
**Quality/standard of peer review**	7.5	27.2	45.6	12.5	7.2	710	2.85
**Speed of peer review**	26.7	25.8	26.1	15.0	6.3	712	2.48
**Post-publication commenting on social media**	3.4	7.8	55.0	23.1	10.7	642	3.30

Country differences are shown in Table 11 as mean values. There are significant differences between countries for all four aspects except for the research integrity where the difference is not statistically significant. US ECRs had the most negative view of the impact on quality and speed of peer review. Malaysian ECRs had the most positive view on all four aspects. The interviews also highlighted the negative impact on the quality of peer review.

**Table 11:** Country differences (mean) for impact of the pandemic on research quality and integrity.

Research quality/integrity aspect	China	France	Malaysia	Poland	Spain	UK	USA	H
**Research integrity**	2.98	2.84	3.41	3.06	2.91	2.88	2.91	11
**Quality/standard of peer review**	2.88	2.67	3.77	2.94	2.90	2.65	2.54	56*
**Speed of peer review**	2.80	2.62	3.50	2.71	2.64	2.43	1.96	73*
**Post-publication commenting on social media**	3.05	3.27	3.68	3.07	3.05	3.38	3.20	16*

*df = 6, p = .001.

### Networking and conferences

With lockdowns, border closures and working from home, one scholarly activity was especially impacted by COVID-19 and that was how researchers interact with one another and network. Conferences and face-to-face meetings played an important role in the pre-pandemic world, but after the pandemic, many conferences and meetings became virtual and physical visits were out of bounds. As Table 12 shows, the overall impact on networking, finding collaborators and maintaining ties with collaborators was more negative than positive. For instance, 67.5% of respondents thought the pandemic had some or significant negative impact on their ability to network. The negative impact was slightly less severe for maintaining ties with collaborators. This might be because finding collaborators purely through online communication might be more difficult than maintaining tiles with existing collaborators. In terms of age, the older the respondent, the more likely they were to consider the impact less negative (Mean ranged from 2.33 for 26–30 years old to 2.88 for 41–50 years old) for all three items (*p* < 0.05).

**Table 12:** How has the pandemic affected your ability to do the following? (%).

Networking	Significant negative impact	Some negative impact	No impact	Some positive impact	Significant positive impact	Total	Mean
**To network**	35.8	31.7	12.2	12.3	8.1	780	2.25
**To find collaborators**	22.0	36.1	21.5	12.7	7.6	772	2.48
**To maintain ties with collaborators**	17.6	38.5	23.0	13.3	7.6	774	2.55

There were significant differences for all three items between the seven countries (Table 13). ECRs in the USA and France found the impact in all three items the most negative.

**Table 13:** Country differences for networking (mean).

Networking	China	France	Malaysia	Poland	Spain	UK	USA	H
**To network**	2.45	2.00	3.77	2.71	2.49	2.22	1.79	82*
**To find collaborators**	2.47	2.45	3.90	2.76	2.63	2.56	2.23	49*
**To maintain ties with collaborators**	2.60	2.32	3.86	2.88	2.65	2.44	2.35	43*

*df = 6, p = .001.

Slightly more than half (434, 54.4%) of respondents attended online/virtual conferences multiple times and another 39.2% (312) attended once or twice. Only 6.3% (50) did not attend any online conference at all (Table 14). With so many of ECRs having attended online conferences, we asked them about their preference regarding the future. As Table 15 shows, while slightly more than a third (37.1%) preferred face-to-face, a small majority (57%) preferred to have virtual options available alongside face-to-face conferences (hybrid) and only a tiny number (5.9%) preferred only virtual conferences.

**Table 14:** Have you attended online/virtual conferences during the pandemic?

		Not at all	Yes, once or twice	Yes, multiple times	Total
China	N	1	11	48	60
	%	1.7	18.3	80.0	100
France	N	4	16	19	39
	%	10.3	41.0	48.7	100
Malaysia	N	2	6	22	30
	%	6.7	20.0	73.3	100
Poland	N	2	10	5	17
	%	11.8	58.8	29.4	100
Spain	N	1	19	28	48
	%	2.1	39.6	58.3	100
UK	N	2	13	12	27
	%	7.4	48.1	44.4	100
USA	N	15	127	143	285
	%	5.3	44.6	50.2	100
All	N	50	312	434	796
	%	6.3	39.2	54.4	100

**Table 15:** Would you prefer virtual conferences to continue post-pandemic?

		No, I prefer face to face	Yes, I prefer to have virtual options available alongside of face-to-face conferences (hybrid)	Yes, I only prefer virtual conferences	Total
China	N	22	30	8	60
	%	36.7	50.0	13.3	100
France	N	13	24	2	39
	%	33.3	61.5	5.1	100
Malaysia	N	6	20	4	30
	%	20.0	66.7	13.3	100
Poland	N	2	13	2	17
	%	11.8	76.5	11.8	100
Spain	N	13	31	4	48
	%	27.1	64.6	8.3	100
UK	N	7	17	3	27
	%	25.9	63.0	11.1	100
USA	N	136	141	8	285
	%	47.7	49.5	2.8	100
All	N	295	453	47	795
	%	37.1	57	5.9	100

Table 14 also shows country differences (*X*^*2*^ = 32.2, df = 12, p = .001) for conference attendance. Attendance was lowest in Poland. There were some subject differences for online conference attendance (*X*^*2*^ = 33.2, df = 16, p = .0006) with mathematical sciences, (71.4%), arts and humanities (67.4%) and social sciences (63.8%) with the highest percentage of those who attended multiple times and environmental sciences (15%) and chemical sciences (14.3%) having the highest percentage of no attendances at all. There were also some subject differences for preference (*X*^*2*^ = 33.2, df = 16, p = .007) with face-to-face option being the most popular amongst physical sciences (48.2%) and life/biological sciences (45.6%) and virtual only being the most popular amongst mathematical sciences (10.7%) and social sciences (9.6%). See Table 15. The highest percentage of hybrid preferences belonged to environmental sciences (67.5%). Country differences are illustrated in Table 15 (*X*^*2*^ = 35.4, df = 12, p = .0004). Poland had the highest preference for hybrid which is aligned with the findings of the interviews. ECRs from the USA were more likely to prefer face-to-face only conferences compared to other countries with 47.7% (136) of them doing so.

We were interested in the main reason behind their preferences and asked them further about it in a free text question. From those who opted only for virtual conferences, 35 left comments why they did so. If we put the odd reasons aside such as “*I hate conferences*. *People seem to treat them like paid vacations*”, the main reasons given by this group included cost (20), time (13), health risk (3), travel fatigue (2), social fear (2 from China), accessibility and wider participation, including for the disabled (3), travel restriction (1), and being environment friendly by not travelling (1). However, one might ask why this group opted for virtual only and not for hybrid, which includes virtual options. One participant provided an explanation that might apply to many of the others:

*“Virtual conferences are more ecological (avoid travel)*. *Face-to-face meetings*, *which imply relocation*, *are not always possible to attend*. *My experience with hybrid conferences is not good*, *the technical side has always been neglected*, *the speakers who are in person are not heard completely well*, *it is tiring*. *The visual part is also not guaranteed*, *there is a tendency for people who are online to be forgotten*. *With online conferences technical quality is better assured”* (woman, arts and humanities, Portugal, translated from Spanish).

Those who chose only face-to-face conferences left 223 comments and many of them were criticism of virtual conferences (119). This is because by now most researchers had attended some online conferences and had first-hand experience of how good or bad, they are. They pointed out that they are simply not as good as face-to-face and they have many shortcomings, such as a disengaged audience, being tiring and exhausting, and the difficulty of attending when you have not got away from your work, especially if you are working from home and so on. There are problems win respect to distraction (5) and attention (12) in virtual conferences. The rest of the comments pointed out values of face-to-face conferences, the most important one being opportunity for networking (84). It is simply easier to interact (40), engage (16) and have in-person conversation (40) in face-to-face conferences. People can meet others by chance, or they can make an appointment for one-to-one chat with another researcher. The feedback is immediate and honest. Some respondents also appreciated its travel aspect which is a change of scenery and a vacation from work. For some it seemed necessary to be able to leave the work behind and engage in a conference. As one respondent stated:

*More genuine connections*, *more opportunities to expand network*. *Feels like more of a break from work*, *new scenery—enables increased connection and a feeling of being refreshed upon return*. *Just not the same when sitting at your desk in front of a screen* (woman, health/medical sciences, Canada).

Hybrid was the more popular choice and 340 respondents who chose it left comments about the reason for their choice. The main reason for choosing hybrid was to keep their options open and benefit from the advantages of both online and face-to-face conferences. Many seemed to favour face-to-face in normal circumstances, but they realise they have to make a ‘compromise’ because sometimes they cannot travel (91) for various reasons such as health concern or family commitment, or they need to save time (65) or money (44), and therefore, a hybrid conference enables them to attend more conferences and not to miss out. Comments for hybrid conferences included similar advantages that the other two groups mentioned for face-to-face and online conferences such as saving time (63) and money (50), networking (47), accessibility (42), convenience (20) and flexibility (23). As the comment below indicates, hybrid conferences can accommodate different types of researchers, for instance, those who enjoy travelling and those who do not, those who love face-to-face presentations, and those who prefer to ask questions in a less confronting virtual environment and so on.

*Some academics are extroverts—they need conferences and to lecture in large halls*. *Other researchers are introverts—they need quiet (see Susan Cain’s work)*. *Some are able to blend these skills*. *So*, *hybrid options are preferred*. *A hybrid would segment the two audience—allowing extroverts to network with other extroverts and vice versa for the online participants* (woman social sciences, USA).

### Big changes in practice or behaviour

The last question of the survey was a free-text question that asked, “*if the pandemic has resulted in fundamental and lasting changes in you research-associated behaviour and/or practices*, *what is the single most important change*?”.

The coding of 435 responses to this question revealed a few themes. A considerable number of responses were related to changes in how work is done including working from home/remotely (38) and the increased use of technology in work which has resulted in an increasing reliance on online meeting, conferences, and generally online interactions (42). Most of these comments were either positive or neutral (mentioning these as changes in their practice). Online technology and communication increased the possibility for collaboration (18) and networking (7). Although, the pandemic (and its lockdown) for some meant less time spent on field work or lab-based research (9), it increased innovation and flexibility in research design and method (13). There were also new opportunities in relation to data, such as online data collection (e.g., online interview and survey) (12) or reusing other people’s data or trying to make use of secondary data collected in the process of research.

However, there were also a group Of ECRs who mentioned the loss of face-to-face interaction (24) whether it was in-person meetings or chatting to colleagues where they could bounce an idea for feedback or establish a relationship. In the word of one of the respondents: “*Relying more on virtual networking has increased the breadth of my professional network*, *but limited its depth and sacrificed quality (relationships*, *follow-up*, *rigor*, *etc*.*) in some cases*” (unknown gender, life/biological sciences, USA).

More ECRs mentioned an increase in their productivity (18) than a decrease in their productivity (7). Some researchers had to shift their research focus or topic (11).

The pandemic clearly gave some ECRs a good reason and enough time to reflect on their work. Some were rethinking their career (11) contemplating leaving academia, and for some others, reflection resulted in them seeking more meaning in what they did and as a result they were now prioritizing quality and impact over quantity (13). One respondent said, “*I am more inclined to do meaningful research than just publish papers*” (woman, physical sciences, USA).

The reflection also meant that many ECRs were more mindful of the impact of the work on their personal life and health. Aiming for a work-life balance was mentioned by 28, and there were some others who commented on health-related matters (15) mentioning problems such as frustration, anxiety, stress, burn out, isolation, and fatigue. Some were suffering from loss of motivation (13). The following quotations illustrates how the pandemic made some ECRs more mindful of their work-life balance.

*I used to try to be ultra-productive no matter what*, *sometimes ignoring my own health and personal life to do so*. *The system of academia drives that*. *The pandemic made me realize that there are so many factors out of my control when it comes to how my research unfolds and other aspects of life that should not be ignored or taken for granted*. *I still try to be productive of course and feel I am definitely successful still*, *but I no longer prioritize my research/other work tasks over literally everything else (social life; family; my health etc*.*) like I used to so often pre-pandemic* (woman, health/medical sciences, USA).

Some of the comments were related to the scholarly communication system, such as slow peer review (5), increased open access (6), unethical practices in research (3) and the damage to the public’s trust in science (5). As a respondent said: “*For me*, *the credibility of science has lost tremendous prestige*, *and I no longer see my future in science as significant or meaningful*” (man, social sciences, Germany).

## Discussion

The study found that the pandemic had a major impact on various aspects of work-life and scholarly communication of ECRs and this is aligned with the findings of past studies [e.g., 33–35].

The pandemic has a mixed impact on productivity, but the number of ECRs whose productivity was affected negatively was greater than those who benefited from some increase in their productivity. While slightly more than two-fifths disagreed with the statement about increase in productivity, about 29% agreed with it. Free-text questions also showed that various factors, such as reduced research time, disruption in research activities (lab and field work), working from home and new responsibilities such as caring and home schooling contributed to the reduction in productivity. The disruption in research activity was found in some previous studies such as (31). The negative impact on productivity was related to career prospects which was negatively impacted for the majority of respondents. Reduced productivity meant that they would be disadvantaged in competing for jobs. Some of the negative impact on career was also related to delaying research, and an inability to network and collaborate due to loss of face-to-face interaction. Another related factor was a reduction in research time for 41.8% (as opposed to an increase for 30.4%). Some of the time taken away from research might have been spent on teaching as in many institutions teaching moved online and required more time in preparation and learning. Besides productivity and career prospect, morale was also affected, even more severely than career prospect and the impact was stronger in some countries like the USA.

Those who benefited from the pandemic by increasing their productivity or obtaining better career prospects tended to be from research areas that had some significance for the pandemic, fields such as epidemiology, biostatistics and so on. The fact that a quarter of ECRs did change research focus to do something more pandemic-related indicates that research resources and priorities shifted to pandemic-related fields. The majority of those who made this change benefited from it and most of the benefit was related to increased productivity and impact.

While the the overall impact of the pandemic on career prospects and morale was negative, research creativity was somewhat positively affected for some ECRs. This was probably because many researchers had to rethink their research design to be able to proceed with their research without or with limited field work or with using digital technologies and some had to make do with whatever data they could get their hands on, whether that was secondary data collected in another study or data shared by other researchers.

The majority worked remotely or from home and more than two-thirds of those who worked form home benefited from it (the negative impact was for a small minority). Men, perhaps not surprisingly, were more likely not to work from home.

The pandemic also caused some changes in scholarly communication behaviour and practices of ECRs. As researchers moved to off-campus working remotely, the use of various means to find and access full-text articles increased. This included both the use of libraries’ online databases and the use of sites such as ResearchGate, Google Scholar and Sci-Hub. The use of Smartphones for searching and accessing articles also increased.

The sheer majority of ECRs have experienced attending virtual conferences and while virtual or hybrid conferences have been embraced by the majority of ECRs, about a third still prefer face-to-face only. While the expanded virtual networking and communication seems to be good for maintaining ties with existing collaborators and partners, it does not make it easy for ECRs to find new collaborators and partners.

The pandemic resulted in more time spent on activities to increase visibility and on prioritising publishing in journals with faster processing speed. Those with caring responsibilities put more emphasis on such activities and this might have been because this group tended to be older, and it is possible that publishing for tenure carries a higher priority for them compared to their younger colleagues. While the priority of such activities increased for some ECRs, respondents that the pandemic had adversely impacted the quality and speed of peer review.

We know from past studies that ECRs are supportive of open access in principle [36], but there seems to be a gap between intention and action in respect to OA as they do not practice OA publishing as much, partly because the fees (APC) are a significant barrier for them. The uptake of preprint repositories was also low.

The questionnaire was intended to take the main findings/highlights from the three rounds of interviews to a larger, global audience in order to determine whether there is more widespread support for them. However, the questionnaire population is demographically different from the interview one, with the latter notably older, more woman, more social science and more skewed towards the United States so we shall not labour the differences, for it is difficult to make comparisons. Nevertheless, it is worth pointing out, in no particular order, the main areas of agreement:

- The pandemic has had the greatest negative impact on China.
- The significant and growing use of Sci-Hub.
- An increase in digital visibility enhancing efforts, often utilising general social media platforms for this purpose.
- The widespread dislike of article processing charges (APC) used for publishing in open access journals.
- The big concerns about loss of interaction, mostly face-to-face and therefore, impacting on the inability to network and collaborate.
- The most severe impact of the pandemic was on researcher morale and not so much on productivity.
- The continuing unpopularity of institutional repositories.
- An increasing use of smartphones for scholarly purposes.

The following suggestions should help address some of the challenges that the pandemic has brought about for ECRs and cancel some of the negative impact of COVID-19.

- As productivity has been impacted negatively for many ECRs, it is ever more important to consider contextual factors in recruitment and promotion of ECRs. Inclusion of statements about achievements relative to opportunity and considering contextual factors such as discipline, country and so on are important for fairness and equity.
- While conferences are gradually moving to face-to-face, it is important to keep the hybrid option available as it increases the accessibility of the events for many ECRs.
- Work flexibility should increase as some people benefit from working from home due to a better mental health and increased productivity.

### Limitations

The study had some limitations which should be considered when interpreting and generalising the results. The methods used for the distribution of the survey could potentially result in a skewed sample. We had to rely on publishers to agree to help us and the subject composition of the authors of the publishers who agreed to help might have impacted the disciplinary distribution of respondents as it can be seen in Table 1. Moreover, the distribution by publishers means that the survey was sent to those ECRs who were authors and had already published one or more papers. Other methods such as distribution via social media might have created another type of bias which is skewing towards respondents who use social media. The fact that we used a combination of approaches helped to cancel out some of such potential biases. We were able to track the distribution channels from which each respondent came, and it was clear that social media did not have an impact as less than 10 responses were received via Twitter. The largest number of responses from a single channel was those coming from the banner put on the Wiley digital library (196) which created little bias because these respondents were researchers who viewed the banner on the site while trying to find or read a paper and might not necessarily be an author of Wiley journals. The second largest group came from a few distribution channels deployed in the USA (192) mostly ECRs who were targeted directly via email by the research team. The third group was *PLoS* journals authors (141) which might have contributed to skewed subject distribution. The remaining responses were mostly the result of the distribution done by the research team in their corresponding countries. Another limitation is that given our distribution method, it was not possible to calculate any response rate. The statistical differences between subject categories and countries should be interpreted with caution as the number of responses in these two variables varied greatly.

## Conclusion

The pandemic clearly negatively disrupted many of the research activities of early career researchers. However, the impact has not been wholly negative, and certain fields, research areas and individuals have benefited from being more appreciated and valued, and, in some cases, seeing increased resources, better productivity and greater impact. While the result of enforced/greater remote working has caused difficulties regarding collaboration and networking, the virtual solutions created to address the problems have left researchers with even more opportunities for collaborating and networking and with more flexible working conditions, that can lead to a more productive and healthy work-life.
